# Proposal of a New Porous Concrete Dosage Methodology for Pavements

**DOI:** 10.3390/ma12193100

**Published:** 2019-09-23

**Authors:** Eduardo Javier Elizondo-Martinez, Valerio Carlos Andres-Valeri, Jorge Rodriguez-Hernandez, Daniel Castro-Fresno

**Affiliations:** 1 GITECO Research Group, Universidad de Cantabria, 39005 Santander, Spain; rodrighj@unican.es (J.R.-H.); castrod@unican.es (D.C.-F.); 2 Institute of Civil Engineering, Faculty of Engineering Sciences, Universidad Austral de Chile (UACh), Calle General Lagos, 2060 Valdivia, Chile; valerio.andres@uach.cl

**Keywords:** Urban pavement, indirect tensile strength, compressive strength, permeability, skid resistance, stiffness modulus

## Abstract

Although porous concrete pavement design methods are mainly focused on maintaining high permeability rates in order to improve their ability to manage stormwater runoff, the mixture strength is paramount for its durability and service life. This paper proposes a new mixture design method for porous concrete, named PCD (porous concrete design), derived from the ACI 522R-10 and ACI 211.3R-02 standards. The aim is to improve mechanical strength in porous concrete mixtures, while ensuring enough permeability for its use in urban roads. With PCD methodology it is possible to obtain mechanical strengths 30% higher than those produced with ACI methodologies, while maintaining permeability rates close to 2 cm/s, lower than those obtained with ACI methods but still enough to manage extreme storm events. Finally, with the analytical Hierarchy Process (AHP) multi-criteria decision-making methodology and also bearing in mind safety variables, the best porous concrete mixtures are the ones produced with PCD methodology.

## 1. Introduction

Due to the development of cities, the population requires more facilities, which leads to more construction: new roads, new buildings, and more blocked soil interrupting the natural water cycle [1]. This is causing many environmental issues due to the groundwater decrease, not to mention the safety and health issues people are exposed to, such as floods, non-point pollution effects, heat island, and other climate change issues [2,3,4,5]. Consequently, porous pavement materials have been studied for many years with the main objective of implementing them on a city scale in order to minimize the environmental impact related to stormwater management caused by urban surfaces [1,6]. There are various types of porous pavement materials, of which the most common and widely studied ones are porous asphalt and porous concrete (PC) pavements [7,8].

Despite the multiple environmental advantages PC present, pavement constructors still do not rely on it, probably due to the reduced lifespan shown by this material. This is mainly due to its physical characteristics, with an open graded aggregate skeleton designed to maintain high air void (AV) content, usually over 20%, which results in low mechanical resistance and reduced service life.

Many researchers have pointed out the lack of fine aggregate or sand in the mixture as the main reason for PC’s poor mechanical capacity [9], changing the structural scheme from continuous material to discontinuous. Consequently, different kinds of additives are included in the mixture to compensate for this loss of mechanical capacity [10,11,12,13,14,15]. With the same objective, other authors have also tried to replace coarse aggregate (CA) with stronger polymers [16], including cellulose fibers as reinforcement [17,18,19,20], and complementing cement with silica fume or fly ash [10,16,21].

However, it is possible that just by changing the dosage method, the mechanical characteristics of porous concrete could improve, while maintaining sufficient permeability for use in permeable pavement systems (PPS). In order to prove this hypothesis, a modified methodology, named PCD (porous concrete design), derived from ACI 522R-10 [22] and ACI 211.3R-02 [23], is proposed and evaluated in this paper. In addition, the analytical hierarchy process (AHP) multi-criteria decision-making methodology [24,25] has been implemented in order to make a comparison between the mixtures and methodologies, considering mechanical, hydraulic, and safety issues, such as skid resistance.

## 2. Proposed Methodology

### 2.1. Discussion of ACI Methodologies

The first step was to compare the two ACI methodologies, where, despite designing in a distinct way, the final mixture dosages are very similar, varying cement paste components by minimum quantities, always in a greater amount for ACI 522R-10. The same amount of coarse aggregate and sand were used for both methodologies. The main difference between the two ACI methodologies is the way to start the mixture design. ACI 522R-10 starts by calculating the components’ weights, calculating the volumes afterwards, as appreciated in [22]. ACI 211.3R-02 starts with the volumes and finalizes with the components’ weights, as stated in [23].

Consequently, ACI 522R-10, considering a well-compacted mixture due to the mechanical press used for compaction, was selected as ACI reference to compare with the newly proposed methodology, mainly because the norm is newer than ACI 211.3R-02 and the proportions were considered to present a more workable mixture, due to the amount of cement paste.

### 2.2. PCD Methodology Description

The proposed porous concrete design (PCD) methodology is based on fixing the target void ratio in compacted porous concrete mixtures, and dosing the raw materials focusing on the cement mortar strength and the voids in compacted mineral aggregates. Consequently, the PCD methodology starts with a proposed s/c and w/c ratio, as well as an AV design. According to the literature reviewed, a w/c ratio in the range of 0.30–0.40 [11,13,26,27,28] and an AV design around 20% [29,30,31] are recommended. CA density was calculated both compacted and uncompacted with EN 1097-3 standard. This led to a compaction parameter, the aggregate porosity (AGP), which aided in calculating the amount of mortar in the mixture. For the aggregate gradation of 8–12 mm employed, the compaction parameter was between 34.64% and 47.99%. Choosing a number between these limits, in this case 44.30%, the amount of CA can be calculated, as seen in Equation (1).

(1) $$\mathrm{CA}=\frac{\left(\delta agregate\right)\left(V_{Tot}\right)\left(100-AGP\right)}{100}$$

where, by subtracting the AGP (in %) from 100, the total percentage of CA can be obtained. Then multiplying it by the total volume of mixture (*V_Tot_*) and the aggregate density, the weight is established. Next, as seen in Equation (2), the difference between the AGP and the AV will give the proportion of mortar, in percentage. Multiplying it by the *V_Tot_*, the mortar’s weight can be obtained.

(2) $$Mortar=\frac{\left(V_{Tot}\right)\left(AGP-AV\right)}{100}$$

The mortar’s weight (MW) can be obtained by calculating its density and multiplying it by the mortar volume. This data enables the calculation of the cement (C), sand (S), and water (W) the mixture will have, with the use of Equations (3), (4) and (5).

(3) C=MW1+wc+sc

(4) S=(sc)(C)

(5) W=(wc)(C)+[(%abs)(CA)+(%abs)(S)]

A relationship between the AV and the amount of cement is calculated, where, when the cement amount is proposed in order to evaluate its influence in the mixtures, the AV tends to increase or decrease, depending on the quantity of cement. Equation (6) demonstrates this.

(6) $$\mathrm{AV}=\frac{\left(V_{TM}-V_{M}\right)\left(100\right)}{V_{Tot}}+20$$

The constant 20 represents the recommended proportion of AV, in %, according to the literature reviewed. *V_TM_* refers to the total amount of mortar in the mixture, considering AV of 20% and subtracting it from the AGP. *V_M_* is calculated by dividing the MW and its density. In this case, the MW is the result of the sum of cement, sand, and water. For the calculation of the real porosity (P), once the mixture is elaborated, Equation (7) is employed:

(7) $$P=\frac{V_{Tot}-\left[(W_{DRY})\left(\frac{{\%}_{CA}}{\rho_{CA}}+\frac{{\%}_{S}}{\rho_{S}}+\frac{{\%}_{C}}{\rho_{C}}\right)\right]-\left[\left(W_{DRY}\right)W_{DRY}\left(\frac{{\%}_{W}}{\rho_{W}}\right)\right]}{V_{Tot}}\left(100\right)$$

where *W_DRY_* corresponds to the mixtures’ weights, in grams, under dry conditions. %*_CA_*, %*_S_*, %*_C_*, and %*_W_* represent the percentage from the total mixture of CA, sand, cement, and water respectively. *ρ_CA_*, *ρ_S_*, *ρ_C_*, and *ρ_W_*, represent the density, in gr/cm^3^, of the mixture components mentioned before.

**Figure 1:**
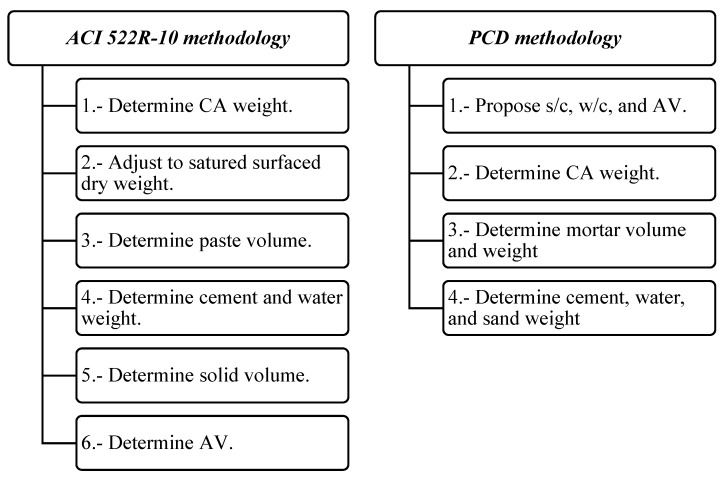
Comparative dosage steps of the ACI 522R-10 and porous concrete design (PCD) methodologies.

**Figure 2:**
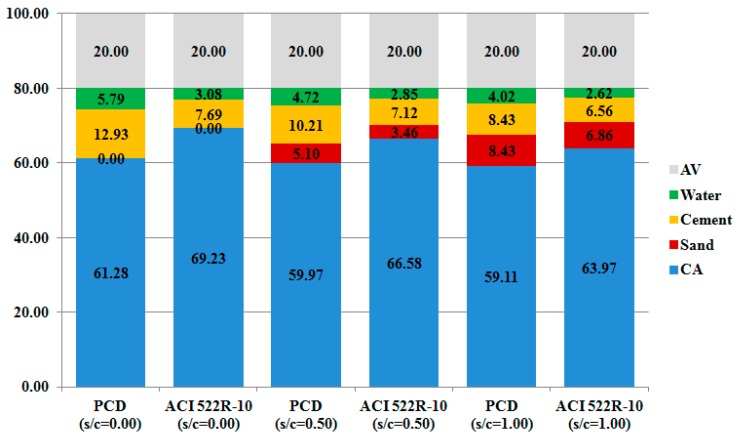
ACI 522R-10 and PCD methodologies dosages according to the s/c.

Figure 1 shows the main steps of both ACI 522R-10 and PCD methodologies. It is expected that PCD mixtures have less CA and more paste content than ACI 522R-10, making it possible that the particles in PCD mixtures achieve better adhesion, increasing the mechanical capacity of the mixtures while having enough permeability, as represented in Figure 2.

## 3. Materials and Methods

### 3.1. Materials Components and Fabrication

To make the porous concrete samples, Portland Cement CEM I 52.5R UltraVal, with a specific weight of 3.144 gr/cm^3^ was used as cementitious material. Porphyric material was used as aggregate, using two gradation sizes: 8–12 mm and 0–2 mm. A w/c ratio of 0.40 and s/c ratios of 0, 0.50, and 1 were employed in order to compare the design methodologies.

Specimens were cylindrical in shape, with 101.60mm diameter and 65 mm height, made in Marshall Molds and compacted with a mechanical press. Fresh mixtures were supplied based on their bulk density and compacted until reaching the target specimen height (65 mm) according to the target void ratio (20%). The whole compaction procedure was electronically controlled in order to fix the target specimen height.

### 3.2. Mixtures Dosages

Three different mixtures varying the s/c ratio were elaborated for each methodology. In addition, the ACI 522R-10 norm states that for mixtures with sand, cement paste content should be reduced by 2% for every 10% of sand of the total amount of aggregate for well-compacted concrete; and by 1% for every 10% of sand, when it is for slightly-compacted concrete. Since these specimens were compacted in a mechanical press, with a controlled load of 200 N/min, well-compacted concrete was considered. The mixture proportions are shown in Table 1. The number in the mixtures’ name corresponds to the s/c ratio employed.

As the ACI-522R-10 norm does not employ the s/c ratio, but a percentage of sand according to the CA amount, the ratio was calculated and the percentage of sand was established for the mixtures of this methodology, as seen in Table 2, derived from Table 6.1 located in the ACI 522R-10 norm [22].

### 3.3. Test Methods

**Figure 3:**
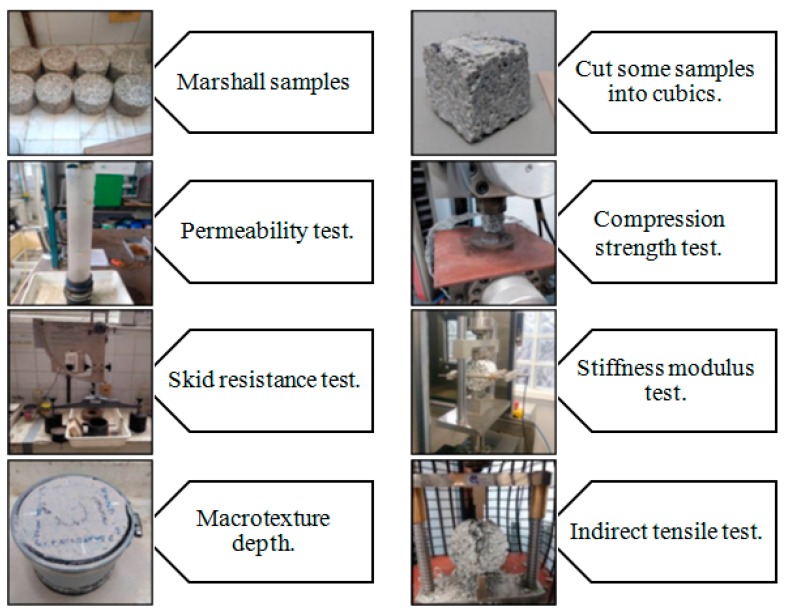
Scheme of research to perform the present investigation.

Figure 3 shows a summary of what was done to carry out this research once the mixtures were made, illustrating the tests performed.

#### 3.3.1. Permeability

The falling-head permeameter (FHP) was used to measure and compare the hydraulic capacity of PC mixtures produced with ACI 522R-10 and PCD methodologies. It consisted of a PVC mold with a 4” diameter to cover the sample. It included a rubber sheet to ensure the sample peripheral waterproofing. The samples were tightened inside the molds with metal clamps. On top of the mold, a methacrylate tube was placed, where the water was filled to start the test. The methacrylate tube was calibrated in order to have a starting and an ending point when the water level reached them. These points were separated 20 cm and a chronometer was employed to measure the time water takes to get from point A to point B. Employing Darcy’s law, Equation (8) was used:

(8) $$k=\left[\frac{\left(A_{sample}\right)\left(h_{sample}\right)}{\left(A_{tube}\right)\left(t\right)}\right]\left[ln\left(\frac{h_{1}}{h_{2}}\right)\right]$$

where k is the permeability coefficient (cm/s); *A_sapmle_* is the area of contact of the sample; *h_sample_* is the height of the sample; *A_tube_* is the area of the tube’s gap; *t* is the time it takes the water to go from the higher point, *h*_1_ to *h*_2_. The heights considered were 21 cm for *h*_1_ and 1 cm for *h*_2_, so the division was not by zero.

#### 3.3.2. Indirect Tensile, Compressive, and Stiffness

The indirect tensile (IT) test was employed in order to understand the behavior of the pavement when vehicles transit on it. Norms EN 13286-42 [32], EN 12390-6 [33], and EN 12390-1 [34] explain the IT test and show the equipment and equations for the test. This test consists of applying load along the cross section of the sample, causing a perpendicular deformation in it, leading to failure.

In addition, some specimens were later cut into cubical shapes of 1:1:1 proportions (6.50 cm × 6.50 cm × 6.50 cm) with a mechanical saw to fulfill the requirements of EN 12390-3 [35] and evaluate the compressive strength (CS) values, despite not fulfilling the minimum dimensions for the norm. Nevertheless, this enabled a comparison to be made between the mixtures and methodologies according to the purposes of this investigation. The same device used for the IT test was employed for this test.

The stiffness of the specimens was measured through the four-point bending test described in the norm EN 12697-26 [36] to evaluate the elastic deformation generated because of the rapid and constant loads pavements are subject to due to traffic. The higher the stiffness modulus, the better the mixture’s resistance to deformation is. It was considered interesting to evaluate which dosage methodology resists the greatest deformations to obtain an idea of the time when cracks will appear in the pavement, making it fail.

#### 3.3.3. British Pendulum Test and Macrotexture Depth

The skid resistance was measured with the British pendulum test in order to assess the pavement behavior in safety terms [37]. This test provides a good parameter for the microtexture estimation of the mixtures and it consists of evaluating the friction of a sample with a device that has a calibrated pendulum. When it is dropped, the pendulum starts losing energy during its contact with the sample. The device also has a needle that points to a determined scale (from 0 to 100) showing the British pendulum number (BPN) of the sample. The higher the number, the higher the sample’s friction is. The area of contact was fixed at 7 cm length, enabling a comparison between the samples.

The macrotexture depth (MT) was calculated. First, the sample was weighed under dry conditions; later, silica sand was spread across the surface of the sample and it was flattened to remove the excess amount. Then, the sample, with its surface voids filled with sand was weighed again. This volume of sand is calculated and divided by the surface area of the sample, obtaining the macrotexture depth [38]. According to this characteristic, pavements can be divided into “very fine texture” (depth < 0.20 mm), “fine texture” (0.20 mm < H < 0.40 mm), “medium texture” (0.40 mm < H < 0.80 mm), “deep texture” (0.80 mm < H < 1.20 mm), and “very deep texture” (1.20 mm < H), where H refers to the height. The macrotexture recommended for dangerous curves and heavy rain is “very deep texture” [39].

## 4. Results and Discussion

Table 3 depicts the mean values obtained in every test, as well as the standard deviation (σ) for each mixture.

### 4.1. Porosity and Permeability

When compacting the mixtures in the mechanical press, the ACI 522R-10 mixtures were harder to compact. This means greater amounts of AV because the CA cannot be accommodated in the mold properly, which is a consequence of the methodology of design. In addition, as seen in Table 1, due to the dosage used, the cement paste was drier than PCD, causing the formation of thinner connecting bridges between the CA particles that did not drain and lead to clogging of the AV. All mixtures for the two methodologies were designed with 20% of voids. However, as seen in Table 3, the real AVs obtained in the ACI 522R-10 mixtures were around 8%–10% higher and the PCD, only between 3%–6% higher than 20%, mainly because the aggregate gradation did not allow for proper compaction. This leads to faster drainage in ACI 522R-10 mixtures, but also less strength. The PCD methodology adjusts the mortar and the aggregate to an established AV content, while ACI methodologies determine the AV depending on the amount of paste.

Due to the higher AV percentage, ACI 522R-10 mixtures obtained permeability rates around 40% higher than PCD mixtures. Contrasting behavior can be observed between the two methodologies, since in PCD mixtures the higher permeability rates were for an s/c ratio of 1, while in the ACI 522R-10 mixtures the s/c ratio was of 0. These results could be explained because PCD does not affect the CA dosage calculated and the initial AV, while the use of sand in ACI 522R-10 mixtures affects the amount of CA, decreasing the AV.

Although permeability rates in PCD mixtures were lower than in ACI ones, they are good enough for a PC pavement. Actually, ACI 522R-10 mixtures are very porous; more than expected from the design. Due to the ACI methodology of design, the lack of sand tended to determine the best mixture in terms of hydraulic properties, being around 35% higher than mixtures with fines. On the contrary, the PCD mixture’s permeability capacity tended even to improve when sand was added in the dosage, because the methodology enabled the adjustment of the mortar component’s quantities in order to maintain the same air void design.

**Figure 4:**
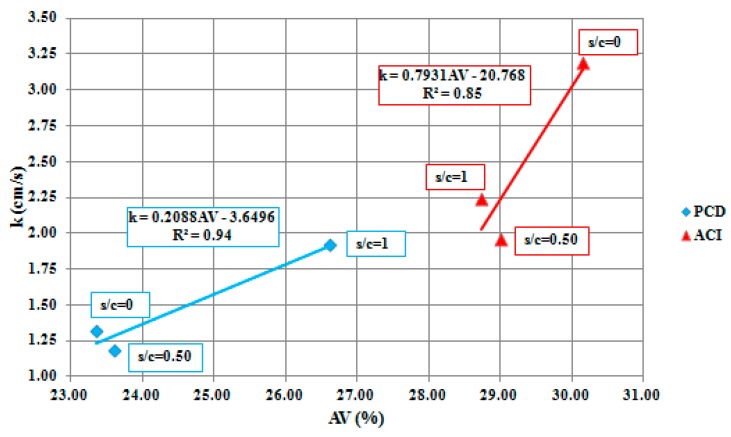
PCD and ACI 522R-10 methodologies linear regression and hydraulic model.

In addition, linear regression was obtained for both methodologies, where the permeability rates can be estimated with the AV design obtained. With this, values of R^2^ of 0.85 for ACI 522R-10 methodology and 0.94 for PCD mixtures were obtained, leading to the conclusion that the modified methodology can help obtain a more accurate hydraulic model when trying to design porous mixtures, as represented in Figure 4.

### 4.2. Indirect Tensile, Compressive, and Stiffness

**Figure 5:**
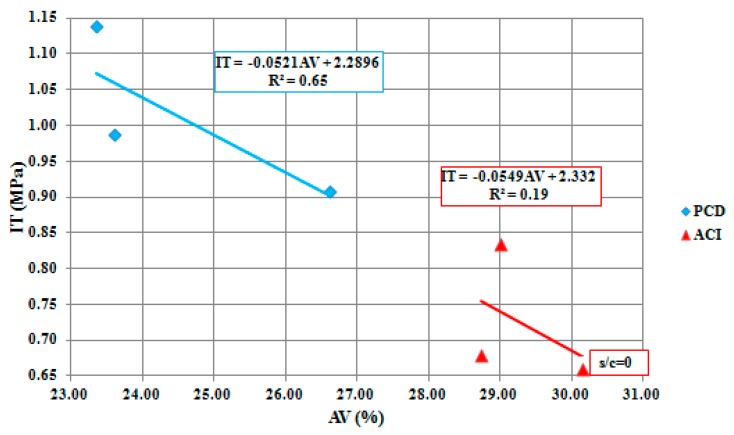
PCD and ACI 522R-10 methodologies linear regression and indirect tensile (IT) model.

Mixtures without sand showed the best IT result in the PCD methodology, with 1.14 MPa of strength. In general, PCD mixtures were around 30% stronger than ACI 522R-10 mixtures, where even the highest value for this methodology was around 9% lower than the worst PCD mixture result. This means that the PCD methodology, when removing the sand from the equation, increases the cement and water amount. Therefore, the cement paste becomes more adhesive and CA particles have better adhesion among themselves. However, permeability can be reduced as the cement paste can turn more fluid and stickier. The model accuracy, as shown in Figure 5, was acceptable in PCD mixtures, with an R^2^ of 0.65 compared to 0.19 in ACI 522R-10, considered to be very low.

**Figure 6:**
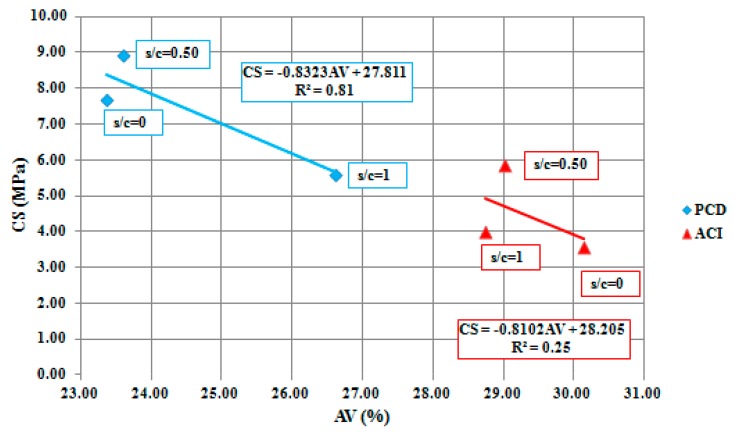
PCD and ACI 522R-10 methodologies linear regression and compressive strength (CS) model.

PCD mixtures obtained around 28% higher CS values than ACI 522R-10 mixtures. The s/c ratio of 0.50 turned out to be the one with the best behavior in both PCD and ACI 522R-10 methodologies. This means that for both methodologies, the addition of sand increases CS. Thus, the more sand, the less cement is used, so the mixture tends to be less adhesive. In ACI 522R-10 mixtures, strength decreases when sand amount is removed, because the same amount of cement is used despite the sand ratio employed. This behavior can be demonstrated with linear regression analysis, where ACI 522R-10 obtained an R^2^ value of 0.25 in relation to the AV obtained in the mixtures, while PCD obtains an R^2^ of 0.81, as demonstrated in Figure 6. PCD mixtures have a well-controlled amount of the mortar, helping to obtain good adhesion between particles.

Constant traffic loads tend to form cracks in pavements. Sometimes these cracks are so small that they cannot be noticed or are located inside the pavement. Nevertheless, these cracks tend to alter the pavement’s behavior and failure tends to follow the crack, getting worse over time because of constant traffic. Therefore, the greater the resistance to deformation, the higher the quality and the longer the lifetime of the pavement. Concrete pavements are well known to be very rigid in comparison with asphalt pavements. Because of the difference in load distribution when vehicles drive over them, concrete tends to distribute loads over a bigger area than asphalt, so deformation is barely noticed. The stiffness modulus is a test employed in asphalt pavements; however, it was considered important to compare the deformation (although minimal) between the methodologies employed, in order to evaluate the lifetime of the pavement.

PCD mixtures demonstrated around 30% more strength under deformation than ACI 522R-10 mixtures, for all s/c ratios evaluated. In addition, it is demonstrated that the addition of sand, in either of the methodologies studied, helps the mixture to become more elastic and resist more deformation force. However, an excess of sand (s/c = 1), tends to decrease this capacity. Nevertheless, the elastic deformation capacity is still around 18% higher than samples without sand, for PCD mixtures, and almost 30% for ACI 522R-10 samples.

### 4.3. Skid Resistance

Skid resistance showed similar results for both methodologies, with the ACI 522R-10 methodology providing slightly higher friction than PCD. In dry conditions, ACI 522R-10 results were around 1.30% higher, and in wet conditions the margin increases to 6.20%. This behavior can be explained due to the high porosity ACI 522R-10 mixtures have, especially in wet conditions, where the water tended to drain very fast and the BPN decreased less than in PCD mixtures. However, all mixtures satisfied the technical specifications of 55–58 for surface layers in dry conditions.

Moreover, it was found that the addition of sand increases skid resistance in PCD mixtures because it tended to make the cement paste rougher, showing the same behavior in ACI 522R-10 mixtures in dry conditions, but the opposite on wet surfaces.

In addition, all mixtures obtained a very deep texture, over 2 mm, where the gradation used is recommended for every type of pavement, especially for dangerous curves and places where it rains a lot. ACI 522R-10 mixture depth was around 6% higher than PCD. Macrotexture is closely related to the AV content. Therefore, when adjusting for the AV content, macrotexture is regulated, achieving better resistance to alterations.

## 5. Comparison

### 5.1. Multicriteria Analysis

For the selection of the best mixture, and therefore, the best design method, the analytical hierarchy process (AHP) multi-criteria decision-making method statistical analysis was employed, as it is one of the most used methods in many fields. The process can be reviewed in [40], and some examples in civil engineering applications are summarized in [25]. This tool allows decisions to be taken by calculating the weights of the results within a normalized matrix, for each test (known as variables). At the end, the weights for every variable are combined for every mixture (alternative), and the one with the highest weight is considered to be the optimal one.

Starting from the scale of comparison proposed by Saaty, employed by many authors [24,40,41,42], a scale of 9 values of importance was proposed to compare the alternatives. This scale is shown in Table 4, where the value is obtained from the pairwise comparison between each alternative in every variable.

The following steps summarize the AHP methodology:

1. Alternatives are placed in an “n” factor matrix and a value of importance is assigned when making the pairwise comparison among the alternatives results. For example, if an alternative “A” has a value of importance of 5 with respect to an alternative “B”, then, “B” will have a value of importance of 1/5 with respect to “A”. That is the reciprocal value. This can be seen in Equation (9):
  (9) A= [a11⋯a1j⋯a1n⋮⋮⋮ai1⋯aij⋯ain⋮⋮⋮a1n⋯anj⋯ann] , aii=1, aij=1aji , aji≠0
2. The matrix is then normalized by dividing each value by the total sum of its column, as demonstrated in Equation (10):
  (10) $$n_{ij}=\frac{x_{ij}}{\sum_{i=1}^{m}x_{ij}};j=1,2,\ldots ,n;i=1,2,\ldots ,m$$
3. The average value in each row in the normalized matrix is then calculated, obtaining a vector for each alternative. The higher the vectors’ value, the better the hierarchy of the alternative among the others.
4. With the use of Table 5, weights are assigned to each variable. A value is designated according to the importance among the variables, and values are placed in a new matrix as Equation (9). Then, step 2 and 3 are repeated, obtaining the variable’s weights.
5. This process is made for each variable (test). Later, each alternative’s value is multiplied by each variable with a weight, w_j_, with the use of Equation (11).
  (11) $$A=\overset{n}{\underset{j=1}{\sum }}w_{j}x_{ij}$$
6. An average value for each alternative in each variable is calculated, obtaining a vector. The vector with the highest value is considered to be the best alternative.

Table 5 shows the relative importance of the variables considered most relevant for evaluation by the authors. For example, for the comparison, permeability was considered to have 0.40 of importance compared to the indirect tensile result, so indirect tensile obtained 0.60 of importance compared to permeability. Mechanical values (indirect tensile and compressive strength) were considered the most important as the main objective is that the pavement resists traffic loads as well as possible. Permeability and skid resistance results were very good in all mixtures, so the importance given was less, but still significant. Elastic deformation was considered the least important of all characteristics as concrete, in general, is very rigid.

Table 6 shows the weights obtained from the AHP analysis, where it can be seen from the average vector column that PCD mixtures are better when trying to achieve a mixture that can fulfill all the characteristics, as they obtained higher values than the ACI mixtures. The ACI-0 mixture, which had no sand, behaves slightly worse than the PCD-1 mixture, but its great permeability helped the mixture to obtain a higher vector at the end.

The best mixture, considering this AHP analysis is PCD-0.5, with the highest vector of all; and the best methodology is the PCD with the three mixtures among the best in contrast to the ACI methodology. In addition, if the importance among the variables of study, presented in Table 5, is modified and all the variables are considered equally important, weights will change. However, the mixtures order of importance remains the same, the PCD methodology proving better than ACI 522R-10.

### 5.2. Viability

As the main problem nowadays with PC pavements is the lack of resistance to handle traffic, in addition to maintaining a reasonable infiltration capacity, it is considered that the PCD methodology is viable due to the increase in the mechanical capacity by about 30%. Despite decreasing infiltration capacity considerably, around 40%, compared with ACI 522R-10 mixtures, PCD permeability is considered to be very good as some authors claimed that permeable pavements should have at least 100 m/day (0.01 cm/s) of infiltration capacity to be functional. However, a simple economic analysis, just considering the materials needed to make 1 m^3^ of PC, is performed to evaluate costs. As seen in Table 7, the results demonstrated that the PCD methodology is more expensive to make, as it needs more cement in the mixture. Some researchers claimed that, in order to increase the mechanical capacity of PC pavements, cement amount should be at least 300 kg/cm^3^ [43,44,45].

When a ratio of s/c = 1 is employed, PCD is 6.25% more expensive than ACI 522R-10. Decreasing the s/c ratio to 0.5, the difference in the cost increases to 10.14%, and when no sand is used in the mixture, the PCD methodology costs 16.11% more. Despite these increments, the range of costs is considered to be within acceptable parameters, according to some investigations [46], and the fact that PCD has a greater long-term durability, due to its strength, leads to the conclusion that it is economically viable. Maintenance is suggested to be the same for both methodologies to maintain a proper function for the PC pavement.

## 6. Conclusions

This paper introduces a modified dosage methodology for porous concrete design (PCD) for use as pavement surface layers with the purpose of balancing the mechanical, hydraulic, and safety properties of mixtures in comparison with the ACI methodologies. The following conclusions can be stated:

- Adding sand in the dosage tends to increase the mechanical capacity considerably in any design methodology. However, while in ACI methodologies, placing sand in the cement paste decreases permeability, it increases permeability in the PCD methodology. This is because in the PCD methodology, the mortar is adapted according to the paste percentage established in order to respect the design ratios (w/c and s/c). Consequently, adding sand to PCD mixtures increases both mechanical and hydraulic properties, up to a limit of sand (s/c = 1).
- Compared to the ACI 522R-10 methodology, the PCD methodology tends to increase mechanical capacity of mixtures (30% higher than those produced with ACI methodologies), maintaining good values of permeability (rates close to 2 cm/s). PCD mixtures tend to resist the impact of traffic loads better and they take longer to form cracks in their structure because of the higher deformation resistance due to the dosage design, where as the paste is less dry and the mixture has greater workability. PCD mixtures obtained a better adhesion between particles.
- Skid resistance results were very similar in both methodologies. Under dry conditions, behavior is practically the same. However, under wet conditions the BPN increases for ACI 522R-10 mixtures. This happens because of the fast permeability of ACI 522R-10 mixtures.
- Vector values were higher in PCD mixtures according to the AHP multi-criteria decision-making method, concluding that this methodology provides better properties than the ACI-522R-10 norm.
- Macrotexture depth results were not considered in the multi-criteria analysis as they do not represent significance in PC pavements behavior. This test was performed to establish a recommended use, explained in Section 3.3.3.
- PCD mixtures could be around 6%–16% more expensive than ACI-522R-10 mixtures (depending on the s/c ratio employed). This is mainly because of a greater amount of cement being employed. However, the PCD methodology is considered to be viable as the long-term resistance of these mixtures is greater.

Future research should be carried out in this line of investigation in order to confirm the advantages and viability of the PCD methodology. The research may include: (1) Implementation of different aggregate gradations and w/c ratios to understand the mixtures’ behavior with the methodology, evaluating mechanical, hydraulic, environmental, and safety characteristics; (2) Implementation of the PCD methodology replacing the cement used as binder with polymers in order to evaluate the use of the methodology with other materials.

## Figures and Tables

**Table 1:** Mixture dosages design for the methodologies in study.

Mixture	w/c	s/c	Aggregate (kg/cm^3^)	Cement (kg/m^3^)	Water (kg/m^3^)	Sand (kg/m^3^)	AV Design (%)
PCD-0	0.40	0.00	1618.33	341.47	152.77	0.00	20.00
PCD-0.5	0.40	0.50	1618.33	275.53	127.35	137.76	20.00
PCD-1	0.40	1.00	1618.33	230.93	118.18	230.93	20.00
ACI-0	0.40	0.00	1889.92	209.97	83.99	0.00	20.00
ACI-0.5	0.40	0.50	1832.65	195.97	78.39	95.14	20.00
ACI-1	0.40	1.00	1775.37	181.97	72.79	190.28	20.00

**Table 2:** ACI percentages of sand and their s/c ratio.

Sand (%)	CA (%)		s/c
	2.36–9.50 mm	4.75–19 mm	
0	99	99	0
5	96	96	0.51
10	93	93	1.12
20	85	86	2.24

**Table 3:** Mixtures general results for every test employed.

Mixture	PCD-0	PCD-0.5	PCD-1	ACI-0	ACI-0.5	ACI-1
AV (%)	23.36	23.60	26.62	30.17	29.01	28.74
σ	0.01	0.01	0.02	0.01	0.01	0.01
k (cm/s)	1.32	1.18	1.92	3.19	1.96	2.25
σ	0.05	0.08	0.02	0.58	0.35	0.55
IT (MPa)	1.14	0.99	0.91	0.66	0.83	0.68
σ	0.05	0.21	0.08	0.19	0.06	0.11
CS (MPa)	7.67	8.93	5.60	3.56	5.86	3.99
σ	0.71	2.07	0.64	0.91	0.55	0.63
Stiffness Modulus (MPa)	11,154	15,757	13,478	6804	11,589	9476
σ	661	904	1227	151	1474	551
BPN (Dry)	62	65	68	65	65	68
σ	9.15	1.27	3.29	0.92	0.41	2.41
BPN (Wet)	52	55	60	62	58	58
σ	2.77	5.48	0.52	0.52	2.74	4.03
MT (mm)	2.86	2.45	3.02	3.52	2.51	2.84
σ	-	-	-	-	-	-

**Table 4:** Values of Importance employed for the Analytical Hierarchy Process (AHP) multi-criteria decision-making method.

Parameters	k	IT	CS	Stiffness Modulus	BPN (dry)	BPN (wet)	Values	
**-**	**Criteria**							-
Equal to	0	0	0	0	0	0	1	
Between	0 and 0.50	0 and 0.12	0 and 1.34	0 and 2238.13	0 and 2	0 and 2.50	2	
Equal to	0.50	0.12	1.34	2238.13	2	2.50	3	
Between	0.50 and 1.01	0.12 and 0.24	1.34 and 2.68	2238.13 and 4476.25	2 and 4	2.50 and 5	4	
Equal to	1.01	0.24	2.68	4476.25	4	5	5	
Between	1.01 and 1.51	0.24 and 0.36	2.68 and 4.02	4476.25 and 6714.38	4 and 6	5 and 7.50	6	
Equal to	1.51	0.36	4.02	6714.38	6	7.50	7	
Between	1.51 and 2.01	0.36 and 0.48	4.02 and 5.37	6714.38 and 8952.50	6 and 8	7.50 and 10	8	
Equal to or greater than	2.01	0.48	5.37	8952.50	8	10	9	

**Table 5:** Importance among variables of study.

Mixture	k	IT	CS	Stiffness Modulus	BPN (Dry)	BPN (Wet)
K	-	0.40–0.60	0.40–0.60	0.70–0.30	0.40–0.60	0.40–0.60
IT	0.60–0.40	-	0.50–0.50	0.95–0.05	0.90–0.10	0.90–0.10
CS	0.60–0.40	0.50–0.50	-	0.95–0.05	0.90–0.10	0.80–0.20
Stiffness Modulus	0.30–0.70	0.05–0.95	0.05–0.95	-	0.30–0.70	0.30–0.70
BPN (dry)	0.60–0.40	0.10–0.90	0.10–0.90	0.70–0.30	-	0.40–0.60
BPN (wet)	0.60–0.40	0.10–0.90	0.20–0.80	0.70–0.30	0.60–0.40	-

**Table 6:** Mixtures’ vector and variables’ weights.

Mixture	k	IT	CS	Stiffness Modulus	BPN (Dry)	BPN (Wet)	Vector
PCD-0	0.046	0.466	0.274	0.087	0.027	0.026	0.037
PCD-0.5	0.033	0.219	0.372	0.464	0.092	0.056	0.038
PCD-1	0.108	0.145	0.085	0.215	0.412	0.214	0.028
ACI-0	0.502	0.030	0.027	0.029	0.092	0.364	0.023
ACI-0.5	0.131	0.097	0.137	0.135	0.092	0.128	0.020
ACI-1	0.181	0.043	0.105	0.070	0.285	0.214	0.021

**Table 7:** Economic analysis between the PCD and ACI 522R-10 methodologies.

Material	ACI-0	ACI-0.5	ACI-1	PCD-0	PCD-0.5	PCD-1
Cement (kg)	43.58 €	40.68 €	37.77 €	70.89 €	57.19 €	47.93 €
Aggregate (m^3^)	56.69 €	54.97 €	53.26 €	48.55 €	48.55 €	48.55 €
Sand (m^3^)	−€	1.90 €	3.80 €	−€	2.75 €	4.61 €
Water (m^3^)	0.16 €	0.15 €	0.14 €	0.29 €	0.25 €	0.21 €
Total	100.43 €	97.70 €	94.97 €	119.73 €	108.74 €	101.30 €
Difference	-	-	-	16.12 €	10.15 €	6.25 €
